# Theory-informed design and dissemination of multilingual menopause CoMICs: the Menopause MEET initiative

**DOI:** 10.3389/fdgth.2026.1813555

**Published:** 2026-07-06

**Authors:** Eleni Armeni, Ugljesa Barac, Esha Kaur, Punith Kempegowda, DEVI Collaboration

**Affiliations:** 1 Department of Applied Health Sciences, School of Health Sciences, College of Medicine and Health, University of Birmingham, Birmingham, United Kingdom; 2 Department of Diabetes and Endocrinology, Royal Free London NHS Foundation Trust, London, United Kingdom; 3 University Clinical Center of Serbia, Faculty of Medicine, University of Belgrade, Belgrade, Serbia; 4 Department of Diabetes and Endocrinology, Queen Elizabeth Hospital Birmingham, University Hospitals Birmingham NHS Foundation Trust, Birmingham, United Kingdom

**Keywords:** comics, education, menopause, multi-lingual, social media

## Abstract

**Objective:**

To characterise patterns of dissemination, including reach, interaction, and sustained engagement over time, and compare how these patterns vary across different social media platforms and linguistic contexts.

**Methods:**

This prospective dissemination and engagement study was conducted at the University of Birmingham between April 2024 and September 2025. Videos on menopause education were co-created using CoMICs (Concise Medical Information Cines) framework by clinicians and patient advocates and translated into 12 languages. The video creation was informed by established learning and implementation theories. The videos were released across YouTube, Facebook, Instagram, TikTok, and X/Twitter. Engagement metrics, including views, likes, shares, comments, and saves, were collected at 3 and 12 months post-release. Analyses of absolute engagement and percentage growth were performed using Python (pandas v2.2, matplotlib v3.9).

**Results:**

Sustained and heterogeneous engagement was observed across languages and platforms. The largest relative increases in views between 3 and 12 months were seen in Georgian (+205%), Gaeilge (+178%), Malay (+160%), Serbian (+128%), and Spanish (+100%). Moderate growth was observed for Portuguese (+43%), Hindi (+27%), and Romanian (+16%). Turkish (+11%) and English (+6%) content showed stable engagement over time.

**Conclusions:**

The Menopause MEET initiative provides a practical framework for scalable, multilingual patient education with sustained global engagement.

## 1 Introduction

Effective patient education is a key determinant of health outcomes, influencing treatment uptake, adherence, and long-term disease management (1). As health information increasingly circulates through digital environments, the dissemination of evidence-based knowledge has become a central challenge in public health (2).

Social media enables large-scale dissemination but introduces variability in engagement driven by platform architecture and user behaviour (3, 4). Evidence suggests that well-designed patient education interventions can improve knowledge, behaviours, and clinical outcomes, yet the mechanisms by which digital content achieves meaningful and durable impact remain incompletely understood (5). Prior work describes both short-lived (“burst”) and cumulative (“long-tail”) engagement patterns, but these have rarely been examined longitudinally across platforms and languages (6–8).

Video-based and multimedia health education interventions have shown promise in improving comprehension, accessibility, and user engagement, particularly among individuals with lower health literacy (9). However, existing approaches are often limited by short-term evaluation, lack of scalability across diverse populations, and insufficient grounding in established theoretical frameworks (10). While some interventions incorporate co-creation or elements of lived experience, there remains considerable heterogeneity in methodological rigour, reproducibility, and conceptual underpinning (11). In addition, engagement is frequently reported using descriptive metrics such as views, without clear differentiation between transient visibility and meaningful interaction (12, 13). Critically, there is limited empirical understanding of how theory-informed design features, platform-specific dynamics, and contextual adaptation interact to influence real-world dissemination outcomes (14). Consequently, it remains unclear which dissemination strategies support sustained engagement and effective knowledge diffusion, and which primarily generate short-term exposure without lasting impact (15).

Existing co-design and co-production models have demonstrated benefits for relevance and acceptability in digital health interventions, but may be resource-intensive, difficult to standardise across multilingual settings, and variably operationalised in practice. Recent work has highlighted methodological challenges in scaling participatory approaches while maintaining contextual relevance and scientific consistency (14, 16–18). The Menopause MEET initiative therefore adopted a hybrid model combining expert-led evidence synthesis with iterative validation by patient representatives and multilingual menopause specialists. This approach aimed to balance scalability, consistency, and contextual adaptation across diverse linguistic settings.

Existing frameworks for developing research-based and evidence-informed media interventions—including co-design, participatory, and theory-informed approaches—provide useful foundations but are rarely integrated or evaluated in multilingual, cross-platform contexts (19, 20). Moreover, the incorporation of lived experience remains heterogeneous and inconsistently operationalised across scalable development pipelines (21). Prior digital health studies have frequently evaluated engagement within single platforms, short follow-up periods, or monolingual populations, limiting understanding of how culturally adapted educational content disseminates longitudinally across diverse audiences (22, 23). Collectively, these limitations highlight the need for theoretically informed, scalable, and culturally adaptive models of digital health dissemination that can be evaluated longitudinally across multiple platforms and linguistic settings.

Menopause is a relevant case study due to persistent information gaps and misinformation, particularly among multilingual populations (24, 25). Despite affecting a substantial proportion of the global population, menopause remains underrepresented in health education strategies and is frequently associated with misinformation and unmet informational needs, particularly among multilingual and underserved populations (25). These challenges are further compounded in the context of hormone therapy, where evolving evidence, changes in clinical guidelines, and persistent legacy safety warnings have contributed to confusion among both patients and clinicians (26). This highlights the need for accessible, evidence-based educational resources that can be delivered at scale and adapted to diverse cultural and linguistic contexts (14). Menopause therefore represents an important test case for scalable multilingual digital health communication.

In this study, we examined the use of social media as a mode for menopause education. Specifically, we: (1) characterised patterns of dissemination, including reach, interaction, and sustained engagement over time; (2) compared how these patterns vary across different social media platforms and linguistic contexts. We then interpreted the findings to examine how theory-informed design features and contextual adaptations may be associated with dissemination outcomes.

## 2 Methods

The Menopause MEET (Myth-busting Education with Engaging Translations) initiative includes the development of a dedicated, multilingual digital platform to address the persistent lack of high-quality visual education in menopause care. This platform hosts evidence-based visual resources, including infographics, short-form videos, and interactive educational modules, designed to enhance comprehension and accessibility across diverse populations. By translating and culturally adapting content into multiple languages, the initiative seeks to broaden global reach, counter misinformation, and improve health literacy among both patients and healthcare professionals.

This prospective dissemination and engagement evaluation study was conducted from April 2024 to September 2025, using the CoMICs framework (27, 28). Table 1 summarises the theoretical frameworks informing intervention design. These frameworks informed decisions on content structure, audiovisual design, and dissemination strategy. The intervention followed five stages: (1) topic identification and script development, (2) video production, (3) multilingual adaptation, (4) dissemination and publication strategy, and (5) impact evaluation.

**Table 1:** Theory-informed design features and dissemination outcomes of the menopause CoMICs.

Theoretical framework	Core theoretical principle	Design feature in menopause CoMICs	Expected outcome	Observed outcome in MEET study
Knowledge-to-action (kta) framework (35, 38)	Knowledge must be adapted to local context and refined iteratively with stakeholders	Co-creation of scripts with clinicians and patient advocates; multilingual translation by local menopause specialists; iterative peer review	Contextually relevant, credible, and accessible educational content	Sustained engagement across multiple non-English languages, with greater long-term growth compared with English content
Cognitive load theory (33)	Learning is optimised when extraneous cognitive load is minimised	Short, focused videos; simplified explanations of physiology; limited on-screen text	Improved comprehension and reduced cognitive burden	Stable engagement and sustained viewing over time, particularly on long-form platforms (YouTube, Facebook)
Cognitive theory of multimedia learning (34)	Dual-channel processing enhances learning when narration is paired with meaningful visual cues	Narrated explanations synchronised with animations and visual prompts	Deeper understanding and improved retention	High engagement efficiency and sharing on platforms supporting audiovisual learning
Diffusion of innovations theory (36)	Adoption depends on perceived attributes of the innovation (e.g., compatibility, usability, accessibility)	Concise, animated, culturally adapted, low-barrier audiovisual format disseminated across platforms	Wider adoption, secondary sharing, and sustained dissemination	Long-tail engagement on Facebook and YouTube; continued growth in culturally adapted language versions
Keller's ARCS model (39)	Attention, relevance, confidence, and satisfaction enhance motivation to learn	Animated storytelling; culturally relevant examples; reassuring tone	Increased motivation and trust in educational content	Prior CoMICs work demonstrated superior DISCERN scores and perceived reliability

### 2.1 Script generation

A pragmatic, structured, literature-informed approach was used to identify commonly asked questions about menopause. Searches were conducted across PubMed, Cochrane Library, and Embase using combinations of Medical Subject Headings (MeSH) and keywords including “menopause,” “frequently asked questions,” “common concerns,” “symptoms,” and “management”, conducted between January and March 2024 and focused on patient-facing educational needs rather than exhaustive systematic retrieval. Retrieved topics were screened for relevance to patient-facing educational needs and grouped into thematic categories by two independent reviewers, with discrepancies resolved through consensus discussion.

A multidisciplinary team comprising endocrinologists, gynaecologists, and healthcare professionals collaborated with representatives from patient support groups to review and refine the identified topics. Topics were independently assessed and refined through consensus discussions to ensure alignment with both evidence-based priorities and lived patient experience. Five overarching thematic areas were identified, each subdivided into 3–5 specific subtopics.

Scripts were developed using a standardised structure that included: (1) definition of the concept, (2) key symptoms or implications, and (3) evidence-based management or advice. Initial scripts were drafted in English through a structured collaborative authoring process involving five study investigators (including two clinicians and three researchers). A lead author generated the first draft, which was iteratively refined through group review and consensus-based feedback, without using generative artificial intelligence or professional scriptwriters. English was used as the source language to ensure consistency in scientific content and to facilitate standardised downstream translation across multiple languages, a common approach in global health communication initiatives (29, 30).

Scripts underwent iterative validation involving two early-career researchers, one consultant endocrinologist, and representatives from patient support groups, with at least two structured review cycles conducted prior to finalisation. Review cycles were conducted asynchronously via shared documents and email-based feedback, with approximately 2–4 rounds per language version. The core research team coordinated revisions centrally and consolidated feedback prior to final approval. Feedback was incorporated through structured review cycles, consistent with Knowledge-to-Action principles emphasising contextual adaptation and stakeholder refinement. Each iteration was assessed against predefined criteria: (1) scientific accuracy, (2) clarity and readability for a lay audience, and (3) relevance to patient concerns. Any disagreements during review were resolved through consensus, with final approval provided by the senior clinical investigator. All content was cross-checked against current international guidelines to ensure scientific accuracy, clarity, and clinical relevance.

### 2.2 Video creation

Video production followed a predefined recruitment and standardisation protocol (27). 10 Undergraduate students pursuing medical qualifications and resident doctors were recruited globally via targeted social media calls (Twitter/X, TikTok, Instagram, YouTube Shorts) and institutional email lists across participating academic centres. Invitations were also extended to members of patient support groups affiliated with collaborating organisations. Participants were selected based on prior engagement with CoMICs or demonstrated interest in medical education. All outputs were assessed against a predefined internal checklist (covering content accuracy, structural consistency, audiovisual clarity, and cultural appropriateness) to ensure consistency in content accuracy, structure, and delivery.

Those selected to contribute to video creation were provided with training consisting of written guidance, example videos, and instructions outlining content delivery, tone, and technical requirements to ensure consistency in format, tone, and delivery. Each volunteer was then assigned a predefined script and instructed to record videos in portrait orientation and under 30 s, consistent with principles of cognitive load reduction and in line with platform-specific requirements. Submissions were completed within predefined timelines, with opportunities for repeat contributions.

Visual elements (images, text overlays, emojis) were selected during post-production by the core research team according to predefined criteria: (1) scientific accuracy, (2) clarity of message, (3) cultural appropriateness, and (4) accessibility for a lay audience, with selection performed centrally using a standardised internal review approach to ensure consistency across all videos.

All videos underwent an iterative, multi-stakeholder review process involving early-career endocrinologists, menopause specialists, and patient representatives. Feedback was provided through structured review cycles and incorporated prior to final approval. Contributions were acknowledged by providing a certificate of participation and/or including their names in the videos as appropriate; no financial compensation was provided.

### 2.3 Multilingual dissemination

Languages were selected pragmatically based on collaborators' linguistic capabilities and the geographic diversity of the research network, prioritising regions with active collaborating clinicians and the potential to reach underrepresented or non-English-speaking populations. Analytics were available for 10 language versions and included in the present analysis. In addition, one author (EA) undertook targeted outreach by contacting senior chairs of European and international menopause societies to present the programme and invite broader participation, thereby further enhancing international engagement and dissemination.

Menopause specialists were identified and were invited to participate via direct communication through existing academic networks, collaborating institutions, and outreach to national and international menopause societies. Those expressing interest in collaborating did so voluntarily; no compensation was provided for their contributions. Engagement involved iterative asynchronous review of translated scripts and videos, with feedback provided through structured review cycles coordinated by the core research team. Translations were performed by native-speaking menopause specialists and coordinated through the respective national menopause societies, with at least one additional clinician review where feasible. Feedback was incorporated through iterative review cycles to ensure linguistic accuracy, clinical validity, and cultural appropriateness. Voiceovers were recorded and synchronised with the original videos to produce multilingual versions. Each translated video underwent a further validation step to ensure cultural appropriateness and fidelity to the original content.

Social media platforms (YouTube, Facebook, Instagram, TikTok, and X/Twitter) were selected as dissemination channels for their scalability, global reach, and ability to facilitate both primary dissemination and secondary user-driven sharing. Engagement was assessed using platform-specific metrics, including views, watch time, audience retention, likes, shares, and comments, which reflect both reach and interaction (31).

Dissemination was standardised through a release schedule and platform use but not controlled beyond initial publication, allowing observation of organic engagement patterns. No paid promotion, influencer partnerships, or algorithmic boosting strategies were employed.

### 2.4 Content publication

The finalised CoMICs were released on alternate days between 1 and 18 October 2024 on YouTube, aligned with World Menopause Day. Videos were titled according to their subtopics and accompanied by relevant hashtags to optimise visibility.

Content was disseminated across multiple platforms via central project accounts. Volunteers were not proactively tagged in shared content; instead, they were provided with links and could choose to share or tag themselves, depending on their preferences and platform use. A central website hosting all multilingual videos was created to facilitate access (https://sites.google.com/view/menopausecomics/home). Collaborating societies and organisations were also invited to share content within their own networks, resulting in a hybrid dissemination model combining centralised publication with decentralised amplification.

### 2.5 Impact evaluation

Engagement data were extracted from platform-level analytics at two time points: 3 months (January 2025) and 12 months (September 2025) post-publication. Metrics included total views, likes, shares, comments, saves, and changes in follower/subscriber counts.

Engagement was conceptualised as both reach (views) and interaction (likes, shares, comments) (32). This approach reflects established social media analytics frameworks, which distinguish between passive exposure (reach) and active user engagement (interaction), and emphasise the importance of normalising interaction relative to exposure to enable comparison across platforms with differing visibility dynamics. Engagement efficiency was defined as the proportion of interactions relative to total views, providing a normalised measure across platforms with varying exposure levels (31). This operationalisation is consistent with established social media analytics frameworks that conceptualise engagement as interaction relative to exposure.

Analyses were descriptive, focusing on absolute engagement and percentage growth over time across platforms and languages. No inferential statistical testing was performed, as the study aimed to characterise dissemination patterns rather than evaluate causal effects. This approach aligns with the study's aim to characterise real-world dissemination dynamics rather than test predefined causal hypotheses.

### 2.6 Ethical consideration

The study involved secondary analysis of anonymised, non-identifiable data and did not include human participants or individual-level interventions. Therefore, formal ethical approval was not required under applicable institutional and research governance guidelines. Furthermore, although content was disseminated via commercial, profit-generating social media platforms, no monetisation strategies (e.g., advertising revenue, sponsored content, or influencer partnerships) were employed.

### 2.7 Statistical analysis

Social media engagement metrics (views, likes, shares, comments, and bookmarks) were extracted from platform-level analytics (YouTube, TikTok, Instagram, Facebook, and X/Twitter) and aggregated by language. Engagement efficiency was calculated as interactions relative to total views, as defined above. Absolute engagement counts and percentage growth between the two timepoints were calculated for each platform and language. This operationalisation aligns with prior digital health and social media analytics studies that conceptualise engagement as interaction relative to reach.

Descriptive comparisons were performed to characterise dissemination patterns across platforms and languages. Data were visualised using line charts to illustrate cumulative engagement over time and bar charts to compare relative engagement efficiency and proportional growth. No inferential statistical testing was undertaken, as the study aimed to describe dissemination and engagement trends rather than evaluate causal effects. All analyses were conducted using Python (pandas v2.2, matplotlib v3.9).

## 3 Results

### 3.1 Characterising dissemination patterns (reach, interaction, sustained engagement)

Platforms characterised by persistent content visibility exhibited sustained growth (Supplementary Table S1 and Supplementary Figure S1). Facebook demonstrated marked long-tail dissemination (+924%), while YouTube showed continued accumulation (+59% views, >150% shares). In contrast, high-turnover platforms showed limited persistence. X/Twitter declined in views (−20%) and likes (−41%). Instagram and TikTok showed minimal growth, with reduced sharing on Instagram (−70%), indicating short-lived engagement.

### 3.2 Comparing patterns across platforms and linguistic contexts

YouTube and Facebook maintained or improved their absolute engagement counts, though Facebook's engagement efficiency fell as views outpaced interactions. Like efficiency declined on X/Twitter and share efficiency on Instagram, whereas YouTube and X/Twitter improved their share efficiency. TikTok demonstrated moderate reach but lower interaction (Supplementary Table S1, Supplementary Figure S2).

Language-specific patterns varied. Serbian and Spanish showed sustained growth (>100%), while Georgian, Gaeilge (Irish), and Malay demonstrated delayed but substantial uptake. English and Turkish showed minimal growth, suggesting early saturation (Supplementary Table S2; Supplementary Figures S3, S4).

## 4 Discussion

This study contributes to the digital health education literature by presenting a scalable framework for multilingual dissemination of evidence-based menopause education across multiple social media platforms. The findings demonstrate sustained but heterogeneous engagement patterns over 12 months, with marked differences across both platforms and linguistic contexts. Together, these results suggest that long-term digital dissemination is shaped not only by content design, but also by platform architecture, audience behaviour, and cultural adaptation.

The intervention also provides a pragmatic model of co-production that integrates expert-led content development with iterative validation by patient representatives and multilingual menopause specialists. This hybrid approach may offer a scalable alternative to resource-intensive co-design models, particularly in multilingual digital health contexts. The stronger long-term performance of several non-English language versions further highlights the importance of culturally adapted dissemination strategies when engaging diverse populations.

The dissemination patterns observed across platforms and languages can also be interpreted through the theoretical frameworks underpinning intervention design. Design features informed by Cognitive Load Theory and the Cognitive Theory of Multimedia Learning (33, 34), such as short, structured videos with synchronised audiovisual elements, were associated with sustained engagement on platforms that support repeated exposure. Similarly, the Knowledge-to-Action framework is reflected in the enhanced performance of culturally adapted, non-English content, underscoring the importance of contextual adaptation for effective knowledge translation (35). Patterns of long-term accumulation and secondary sharing are also consistent with Diffusion of Innovations theory (36), suggesting that accessibility, simplicity, and cultural compatibility facilitate broader adoption over time. Together, these findings support the internal coherence of a theory-informed approach to digital health dissemination and provide empirical grounding for its application.

This study extends prior work on digital health education, which has largely focused on short-term engagement or acceptability, by providing a longitudinal, cross-platform analysis of dissemination patterns over 12 months (3, 22, 23). It also highlights the value of integrating multiple theoretical frameworks into both intervention design and evaluation, rather than applying theory *post hoc* (19). Further, it underscores the potential importance of multilingual and culturally adapted content in achieving sustained engagement across multiple languages and platforms, an area that is less well studied in digital health research (14, 23, 37).

However, the study relied on platform-level analytics without access to detailed demographic or socioeconomic data, limiting the ability to characterise audience profiles. Dissemination beyond initial publication was not standardised, and variation in secondary sharing across networks may have influenced engagement patterns. In addition, the observational design precludes causal inference regarding the relationship between specific design features and dissemination outcomes. Future studies should incorporate experimental approaches, such as platform-level A/B testing, to more directly evaluate the impact of theory-informed design elements. The use of commercial social media platforms for health dissemination also raises broader ethical considerations beyond formal research governance requirements. Although the present initiative did not involve monetisation, targeted advertising, or the collection of individual-level user data, dissemination through commercial platforms remains influenced by algorithmic visibility, platform governance structures, and audience data practices that are often not fully transparent to researchers or users. Future work should examine how these factors may shape the reach, accessibility, and ownership of digital health communication content across different populations.

## 5 Conclusion

Sustained digital health dissemination appears to be shaped by the interaction of design, platform dynamics, and linguistic context. This study provides a practical framework for developing co-produced, scalable, multilingual patient education strategies.

## Data Availability

The raw data supporting the conclusions of this article will be made available by the authors, without undue reservation.

## DEVI collaboration

Department of Applied Health Sciences, School of Health Sciences, College of Medicine and Health, University of Birmingham, Birmingham, United Kingdom: Bogdan Milenkovic. University Clinical Center of Serbia, Faculty of Medicine, University of Belgrade, Serbia: Bogdan Milenkovic. University of Birmingham Medical School, Birmingham, UK: Shams Ali Baig, Vardhan Venkatesh, Khushi Kumar, and Santhosh Kumar. Menopause Specialist, Cluj Napoca, Romania: Ildiko Bartok. Department of Paediatrics, Obstetrics and Gynaecology, University of Valencia, Valencia, Spain: Antonio Cano. University Hospital Galway, Galway, Ireland: Marina Curran. Clinic NeoLab, Tbilisi, Georgia: Meri Davitadze. Donostia University Hospital, San Sebastián, Spain: Maria del Mar Rubio Arroyo. Department of Obstetrics and Gynecology, Istanbul Medipol University, Istanbul, Türkiye: Fatih Durmusoglu. Department of Obstetrics and Gynecology, Cerrahpaşa School of Medicine, İstanbul-Cerrahpaşa University, İstanbul, Türkiye: Tamer C. Erel and Ipek Betul Ozcivit, Erkan. Women’s Health Development Unit, School of Medical Sciences, Universiti Sains Malaysia, Malaysia: Nik Hussain Hazlina. Poniente University Hospital, Almería, Spain: Alejandra Herrera Munoz. Caucasus Medical Center, Tbilisi, Georgia: Ana Kobaidze. 2nd Department of Obstetrics and Gynaecology, University of Athens, Aretaieio Hospital, Athens, Greece: Irene Lambrinoudaki. Reproductive and Sexual Health Co-Ordinator, Irish College of General Practitioners, Lincoln Place, Dublin 2, Ireland: Deirdre Lundy. Clinic for Endocrinology, Diabetes and Metabolic Diseases, University Clinical Center of Serbia, Faculty of Medicine, University of Belgrade, Serbia: Djuro Macut. Department of Gynecological Endocrinology, Poznan University of Medical Sciences, Poznan, Poland: Blazej Meczekalski. Pantai Hospital Cheras, Kuala Lumpur, Malaysia: Choon Moy Ho. Souza Marques Faculty, Medical School, Rio De Janeiro, Brazil: Leticia Santiago da Silva Ferreira. Department of Gynecological Endocrinology, Poznan University of Medical Sciences, Poznan, Poland: Anna Szeliga. Regina Maria Menopause Center, Cluj Napoca, Romania: Calin Todoran. Department of Obstetrics and Gynecology, OLVG Hospital, Amsterdam, Netherlands: Dorenda van Dijken.
