# Supplementary material for: Theory-informed design and dissemination of multilingual menopause CoMICs: the Menopause MEET initiative
Source: Front Digit Health. 2026 Jul 6;8:1813555. doi: 10.3389/fdgth.2026.1813555 (PMC13381839; doi:10.3389/fdgth.2026.1813555)
Supplement: Supplementary file 1 [file Datasheet1.pdf]

Supplementary Material

Supplementary Table S1. Growth rates per platform (3 months vs 12 months)

Platform	Metric	YouTube	TikTok	Instagram	Facebook	X/Twitter
**Views (3m)**	Absolute number	2133	4802	4989	1417	14890
**Views (12m)**	Absolute number (% change)	3392 (+59%)	5426 (+13%)	5259 (+5.4%)	14508 (+923.9%)	11853 (-20.4%)
**Likes (3m)**	Absolute number	33	11	59	29	99
**Likes (12m)**	Absolute number (% change)	34 (+3%)	11 (0%)	64 (+8.5%)	31 (+6.9%)	58 (-41.4%)
**Shares (3m)**	Absolute number	43	0	20	25	24
**Shares (12m)**	Absolute number (% change)	108 (+151.2%)	2 (N/A)	6 (-70%)	26 (+4%)	34 (+41.7%)
**Comments (3m)**	Absolute number	0	0	5	1	1
**Comments (12m)**	Absolute number (% change)	0	0	5	1	1
**Bookmarks (3m)**	Absolute number	0	0	7	1	2
**Bookmarks (12m)**	Absolute number (% change)	0	3 (N/A)	6 (-14.3%)	1	2

Supplementary Table S2. Language-specific viewership growth (3 months vs 12 months)

**Language**	**Views (at 3 months)**	**Views (at 12 months)**	**Growth %**
Serbian	3522	8032	128.1
Spanish	2862	5730	100.2
Turkish	2438	2694	10.5
English	2042	2154	5.5
Portuguese	652	929	42.5
Hindi	1083	1374	26.9
Romanian	821	952	16.0
Malay	1322	3436	159.9
Georgian	1293	3946	205.2
Gaeilge	1369	3803	177.8

Supplementary Figures

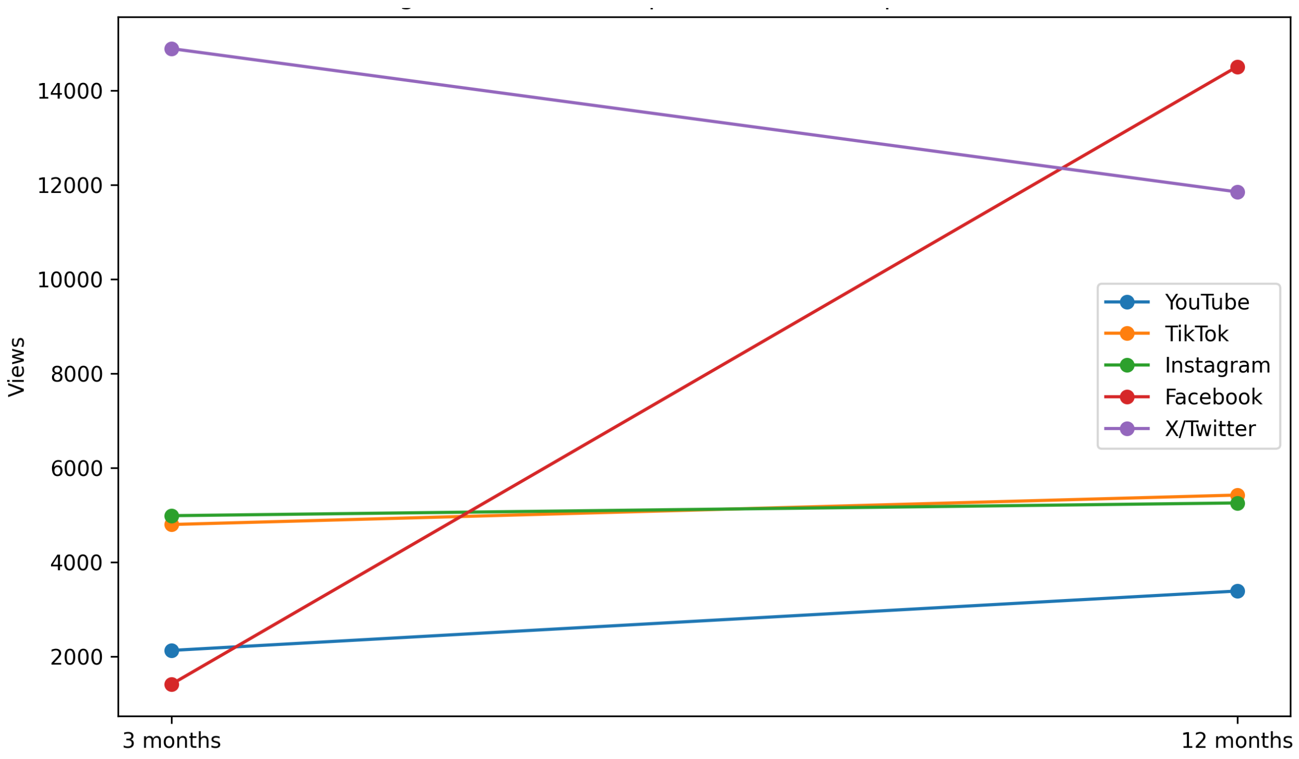

Supplementary Figure S1. Cumulative platform viewership post publication, at 3 months and 12 months. Facebook demonstrated the strongest growth (+924%), surpassing X/Twitter, which declined over time (–20%). YouTube showed steady long-tail accumulation, while TikTok and Instagram plateaued.

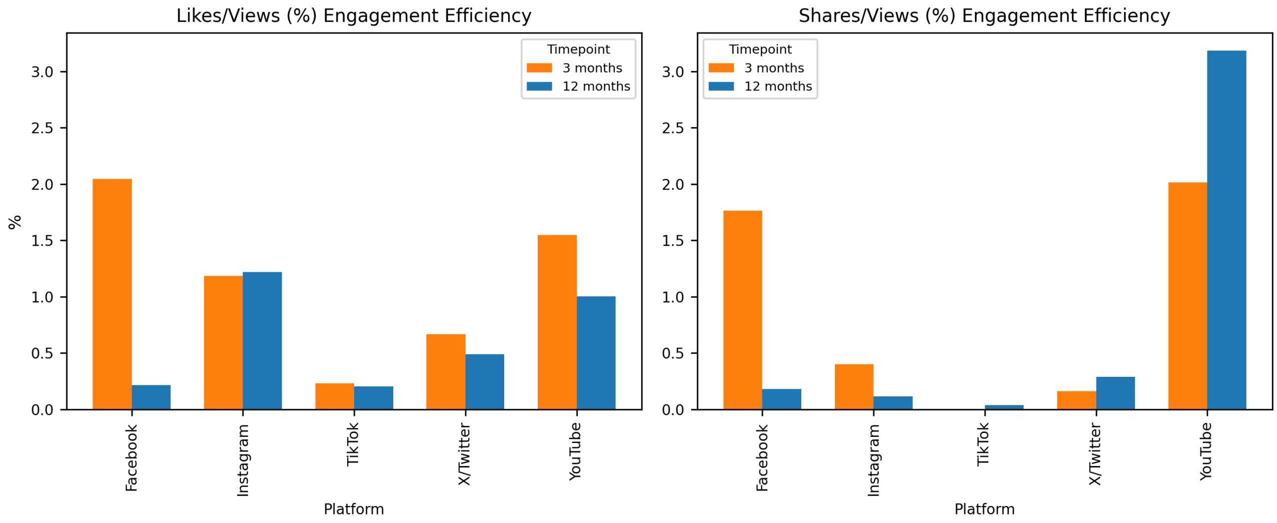

Supplementary Figure S2. Engagement efficiency by platform post publication, at 3 months and 12 months, expressed as likes/views (%) and shares/views (%). Facebook's efficiency fell sharply on both metrics (likes/views 2.0%→0.2%; shares/views 1.8%→0.2%) as views rose far faster than engagement. YouTube and X/Twitter improved their share efficiency over time but saw lower like efficiency, while Instagram held roughly steady on likes and declined on shares.

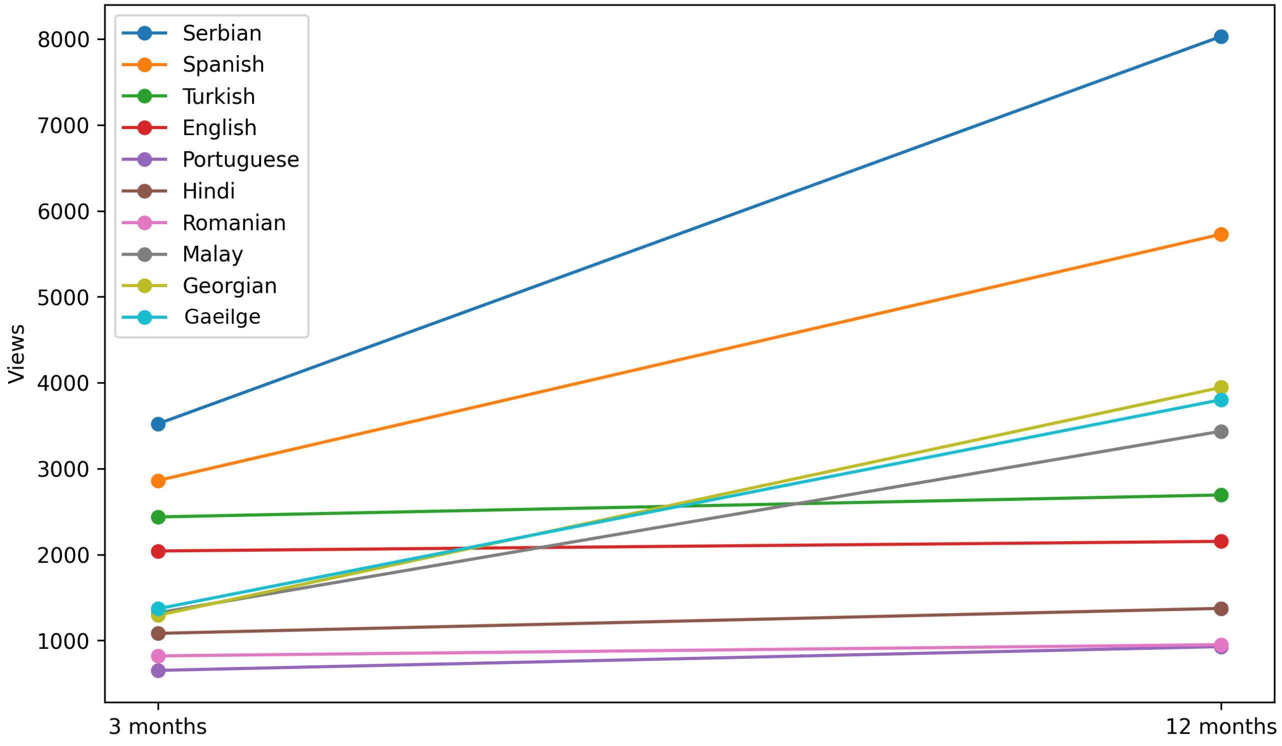

Supplementary Figure S3. Per-language viewership growth at 3 months and 12 months. Serbian, Georgian, Gaeilge, Malay and Spanish content all more than doubled in audience reach, while Turkish and English plateaued.

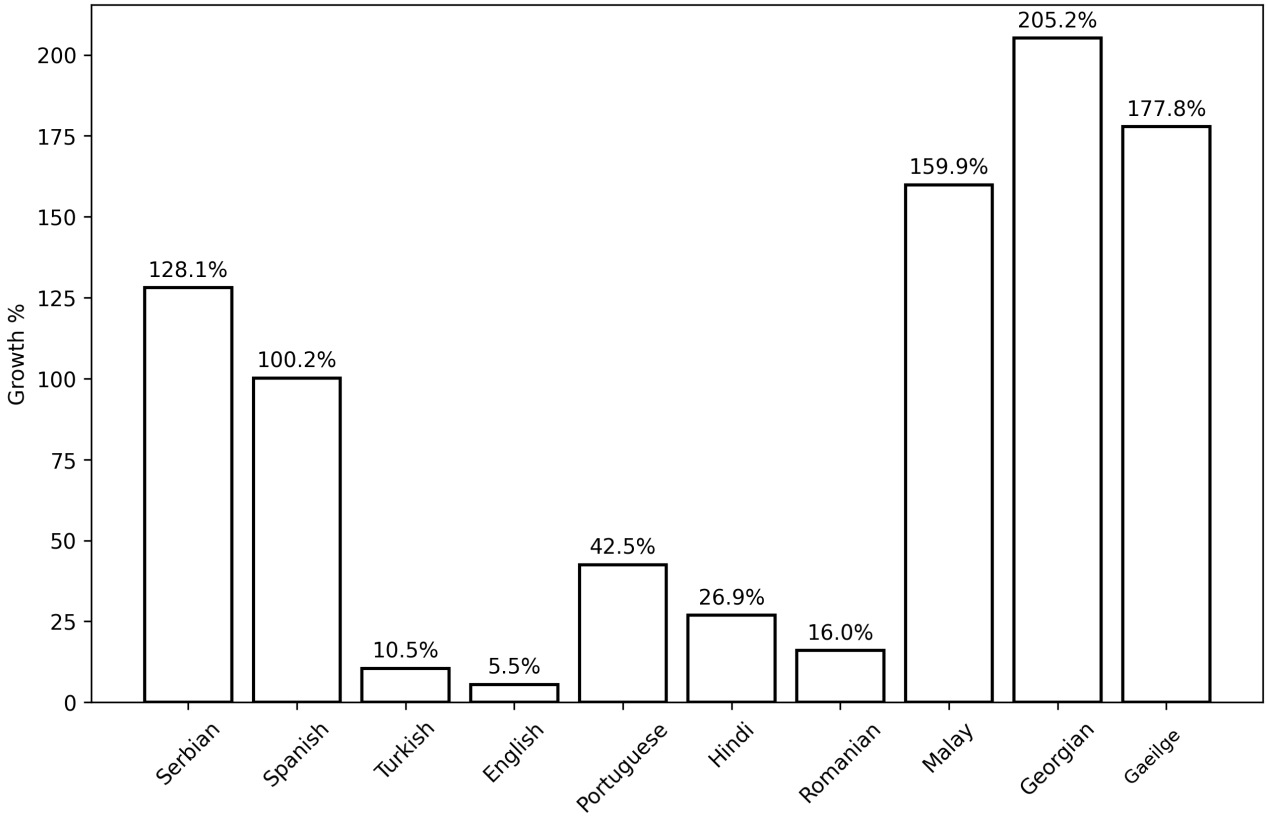

Supplementary Figure S4. Relative growth in viewership (%) per language post publication, at 3 months and 12 months. Georgian (+205%), Gaeilge (+178%) and Malay (+160%) showed the largest gains, followed by Serbian (+128%) and Spanish (+100%); Portuguese showed moderate growth (+43%), while Turkish (+11%) and English (+6%) remained stable.
